# Temperature-dependent development and freezing survival of protostrongylid nematodes of Arctic ungulates: implications for transmission

**DOI:** 10.1186/s13071-018-2946-x

**Published:** 2018-07-09

**Authors:** Pratap Kafle, Stephanie J. Peacock, Sarah Grond, Karin Orsel, Susan Kutz

**Affiliations:** 1 Faculty of Veterinary Medicine, University of Calgary, Calgary, AB Canada; 2 Department of Biological Sciences, Faculty of Science, University of Calgary, Calgary, AB Canada; 3 Department of Ecology and Evolutionary Biology, Princeton University, Princeton, NJ USA

**Keywords:** *Umingmakstrongylus pallikuukensis*, *Varestrongylus eleguneniensis*, Threshold temperature, Degree-days, Freeze tolerance, Arctic, Lungworm, Climate change, Invasion, *Ovibos*, *Rangifer*

## Abstract

**Background:**

*Umingmakstrongylus pallikuukensis* and *Varestrongylus eleguneniensis* are two potentially pathogenic lungworms of caribou and muskoxen in the Canadian Arctic. These parasites are currently undergoing northward range expansion at differential rates. It is hypothesized that their invasion and spread to the Canadian Arctic Archipelago are in part driven by climate warming. However, very little is known regarding their physiological ecology, limiting our ability to parameterize ecological models to test these hypotheses and make meaningful predictions. In this study, the developmental parameters of *V. eleguneniensis* inside a gastropod intermediate host were determined and freezing survival of *U. pallikuukensis* and *V. eleguneniensis* were compared.

**Methods:**

Slug intermediate hosts, *Deroceras laeve*, were collected from their natural habitat and experimentally infected with first-stage larvae (L1) of *V. eleguneniensis.* Development of L1 to third-stage larvae (L3) in *D. laeve* was studied at constant temperature treatments from 8.5 to 24 °C. To determine freezing survival, freshly collected L1 of both parasite species were held in water at subzero temperatures from -10 to -80 °C, and the number of L1 surviving were counted at 2, 7, 30, 90 and 180 days.

**Results:**

The lower threshold temperature (T_0_) below which the larvae of *V. eleguneniensis* did not develop into L3 was 9.54 °C and the degree-days required for development (DD) was 171.25. Both *U. pallikuukensis* and *V. eleguneniensis* showed remarkable freeze tolerance: more than 80% of L1 survived across all temperatures and durations. Larval survival decreased with freezing duration but did not differ between the two species.

**Conclusion:**

Both *U. pallikuukensis* and *V. eleguneniensis* have high freezing survival that allows them to survive severe Arctic winters. The higher T_0_ and DD of *V. eleguneniensis* compared to *U. pallikuukensis* may contribute to the comparatively slower range expansion of the former. Our study advances knowledge of Arctic parasitology and provides ecological and physiological data that can be useful for parameterizing ecological models.

## Background

The extreme climate of the Arctic makes it one of the most challenging environments for living organisms. Winter temperatures can drop to -50 °C in many parts of the Arctic, and sub-zero temperatures last for nearly two-thirds of the year. The summers are short, cool, and dry, providing a narrow developmental window for ectotherms, including parasites, and a short growing season for endotherms and plants [1–3]. Despite these adversities, a diverse group of flora and fauna, ranging from large mammals to microscopic parasites, constitutes Arctic biodiversity [4–6]. Studies have shown that these organisms develop unique physiological and behavioural strategies to cope the extremes [7, 8].

In the animal kingdom, nematodes are considered the most diverse and successful organisms for their ability to adapt to diverse habitats and survive extreme environmental conditions [9, 10]. Nematodes are often used as sentinels of climate-change impacts because larval development inside the intermediate host is temperature-driven and free-living larval stages are influenced by the external environment [5, 11–13]. For instance, lungworm-ungulate systems in the Canadian Arctic have become key in the understanding of the impacts of climate warming on host-parasite systems [5, 14, 15]. In order to determine the current and future impacts of climate change on disease dynamics and ecosystem health, laboratory- and field-based experiments can shed light on the temperature-dependent biology and ecology of both nematode parasites and host species [16–18].

Two lung nematodes, *Umingmakstrongylus pallikuukensis* Hoberg, Polley, Gunn & Nishi, 1995 and *Varestrongylus eleguneniensis* Verocai, Kutz, Simard & Hoberg, 2014, are parasites of Arctic ungulates in areas of the Canadian Arctic mainland and Victoria Island in the Arctic Archipelago [19–21]. *Umingmakstrongylus pallikuukensis* is a host specialist and only infects muskoxen (*Ovibos moschatus*) [22, 23], whereas *V. eleguneniensis* is a multi-host parasite that infects muskoxen, caribou (*Rangifer tarandus*) and moose (*Alces alces*) [20, 21, 24]. The life-cycle of both parasites is indirect and involves a gastropod intermediate host. In the intermediate host, the first-stage larva (L1) develops into a third-stage larva (L3), and the development process is dependent upon temperature. Below a specific temperature (T_0_; lower threshold temperature), larval development inside the intermediate host is minimal, and development to L3 does not take place [25, 26]. Above the T_0,_ L1 develop to L3 after accumulating a certain amount of heat, quantified as development degree-days (DD) [25]. The L3 are transmitted when the definitive ungulate host accidentally ingests while grazing a gastropod containing L3 or L3 that have emerged from the intermediate host and are free-living in the environment [27]. The L3 emergence is an adaptation, particularly common in for northern protostrongylids, that may enable the availability of infective L3 in the environment throughout the winter when the gastropods are unavailable [26, 27]. The developing larvae inside the intermediate hosts are protected from temperature extremes by the thermoregulatory behaviour and, presumably, the freeze tolerance physiology of the gastropods [1, 28]. However, the L1 and emerged L3 are under direct exposure to the external environment and can be exposed to prolonged and intense sub-zero temperatures.

**Fig. 1:**
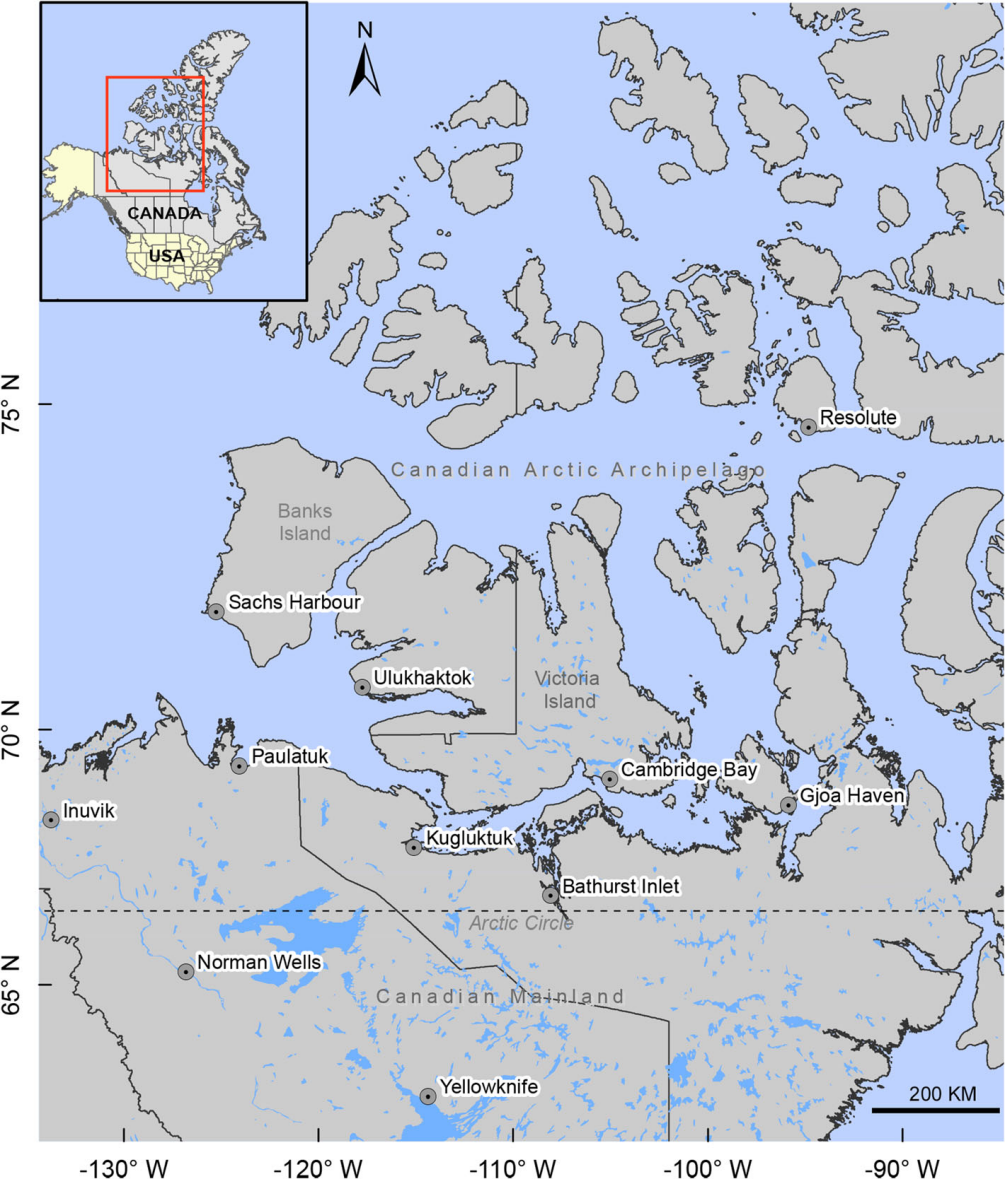
Map showing the arctic mainland Canada and Canadian Arctic Archipelago

*Umingmakstrongylus pallikuukensis* and *V. eleguneniensis* co-occur in muskoxen of the western Canadian Arctic and, until recently, were limited to the North American mainland and had not been found in the Arctic Archipelago. However, in 2008, *U. pallikuukensis*, and in 2010, *V. eleguneniensis*, were reported for the first time on southern Victoria Island, Nunavut, Canada [19] (Fig. 1). Climate warming, with the consequent alteration of a previously unsuitable environment to one that is permissive for development and transmission of these parasites, is suggested as the driver for the invasion and establishment on Victoria Island [15, 19]. Since their discovery on the island, both parasites have rapidly expanded their ranges northward, but at different rates, with *U. pallikuukensis* establishing at higher latitudes prior to *V. eleguneniensis* (Kafle, Kutz unpublished data).

One hypothesis for the differential range expansion of the two parasites is different species-specific thermal requirements and tolerances for the development and survival of their larval stages. Indeed, understanding the developmental responses to temperature in intermediate hosts and freeze tolerance of L1 in the environment is vital for understanding the ecology and transmission dynamics of protostrongylids in general. Kutz et al. [25] studied the temperature-dependent development of *U. pallikuukensis* in its intermediate host, but similar information for *V. eleguneniensis* is lacking. Few previous studies have investigated the freezing survival of L1 of protostrongylids [29–32], and none have investigated the short-term or long-term survival of L1 at extreme sub-zero temperatures.

The objectives of this study were to determine the temperature requirements and tolerances of *V. eleguneniensis* and *U. pallikuukensis.* Specifically, we investigated the temperature-dependent development of *V. eleguneniensis* in a gastropod intermediate host, the meadow slug *Deroceras laeve* (O. F. Müller, 1774), and compared the freezing survival of L1 of *U. pallikuukensis* and *V. eleguneniensis*. This study is essential to our understanding of the thermal ecology of these two emerging parasites in the Canadian Arctic. The resulting data on thermal tolerances provide essential parameter estimates for parasite distribution and transmission models [33] and will ultimately advance our knowledge on Arctic parasitology and parasite invasion.

## Methods

Two sets of experiments were carried out to determine the impact of temperature on development and survival of *U. pallikuukensis* and *V. eleguneniensis*. First, temperature-dependent development of *V. eleguneniensis* in *D. laeve* was determined following the methodology used for *U. pallikuukensis* by Kutz et al. [25]. Secondly, the freezing survival of both species at various sub-zero temperatures was evaluated.

### Temperature-dependent development of *V*. *eleguneniensis*

#### Source of gastropods and larvae

The gastropod intermediate hosts, *D. laeve*, were collected from their natural habitat near Sheep River Provincial Park (50.63°N, 114.67°W) and Sundre (51.98°N, 114.69°W), Alberta, Canada. Refuge traps comprised of dampened cardboard, masonite board, and fabric mats, often baited with grape juice, were used for collecting slugs near creeks and wetlands [34, 35]. The slugs were identified to species by external morphology [36] and transported to the laboratory, where they were stored and maintained in a perforated Rubbermaid® (Rubbermaid, Atlanta, USA) container [25] at 8 ± 2 °C with 12 h light. All the slugs were collected during late spring/early summer and infected within two weeks of collection. The slugs that appeared old and less active were excluded from the the experiments. The infected slugs weighed 104 ± 40 mg (mean ± SD). First-stage larvae of *V. eleguneniensis* were isolated from the feces obtained from wild muskoxen in northern Quebec (58.75°N, 68.55°W) using the modified beaker Baermann technique [37]. The fecal samples had been frozen at -20 °C for over five years and repeatedly confirmed to have only *V. eleguneniensis* L1 [20, 38]. The species identity was reconfirmed by morphology [39] and PCR. L1 were collected in a Falcon tube® (Eppendorf, Hamburg, Germany) stored at 4 °C (12–18 h) before the infection.

#### Slug infection with *V. eleguneniensis* L1

The experimental infection was performed as previously described by Hobert et al. [22] and Kutz et al. [25], with some modifications. Briefly, foot lesions of the wild-caught slugs were checked under dissecting microscope to ensure that slugs were not infected with other protostrongylids [27]. For each of five temperature trials, 35 slugs (40 at 8.5 °C) were used. Slugs were infected in groups of five in a medium-sized (9.1 cm) Petri dish (VWR, Canada). First, the Petri dishes were lined with Whatman®#3 filter paper (GE Healthcare Life Sciences, UK), moistened with clean tap water and 1000 (or 1500 for the 20 °C trial) motile L1 (estimated from aliquot counts, in 2 ml tap water) were spread evenly on the filter paper. The slugs were then placed on the Petri dish to start the infection. Contact between slugs and L1 was ensured using plastic tweezers to gently move the slugs that had crawled on the sides or lids of the Petri dish back to the center of the Petri dish every 15 min for three hours. All infections were performed at room temperature (20 ± 1 °C) for three hours (14:00–17:00 h). For each trial, the dishes were then transferred to the respective temperature-controlled incubators, and infections were continued overnight. At 9:00 h the next morning, the slugs were transferred to a new Petri dish lined with moistened filter paper (except for 8.5 °C trial where slugs were moved to a single larger Rubbermaid® (Rubbermaid, USA) container), and food (clean lettuce, carrot and a piece of chalk) was provided. Slugs were then kept in darkness for the remainder of the experiments, and the temperature was monitored every 15 min (or every one 1 hour in the 8.5 °C trial) using LogTag® temperature recorder (LogTag recorders, NZ).

#### Slug digestion and larval examination

For each trial, three slugs were haphazardly selected at designated days post infection and digested in a pepsin hydrochloride solution [22, 25]. The digestion days were chosen based on previous trials for *V. eleguneniensis* [38], and known development rates for related protostrongylids [25, 26, 38]. The goal was to determine what day the first intermediate third-stage larvae (iL3), defined as a motile larva with fully developed intestinal cells [25], was present. Slug digestions were started at least five days in advance of when the earliest L3 were expected, and the first iL3 were detected at least three days after digestions began. Larvae isolated from the digests were examined under 400× magnification (Olympus CKX41, Olympus, Tokyo, Japan) and the developmental stage was determined [25]. The day when the first iL3 was detected in at least one of the digested slugs was the endpoint for determining development rate, as this was the endpoint used in previous studies on other species [25, 26]. After detecting iL3, the remaining slugs were digested, and L3 quantified, except in the trial at 12.5 °C, where six slugs were separated into individual dishes to do a pilot study on L3 emergence.

### Freezing survival of *U. pallikuukensis* and *V. eleguneniensis* L1

#### Sources of L1

*Varestrongylus eleguneniensis* L1 were obtained from the fresh feces of a captive muskox that was experimentally infected with the larvae obtained from wild muskoxen from northern Quebec (58.75°N, 68.55°W). *Umingmakstrongylus pallikuukensis* L1 were obtained from fresh fecal samples collected from wild muskoxen near Norman Wells, Northwest Territories (63.35°N, 126.52°W). Within 24 h of collection, fecal samples were transported to the lab in whirl packs (Nasco Whirl-Pak, Nasco, Ontario, Canada) maintained at 4 ± 1 °C (temperature monitored by LogTag® temperature recorder) and stored at 4 ± 1.5 °C until processed. For both parasite species, L1 were extracted using the Baermann method [37] within one week of collection. The species’ identities were confirmed morphologically following the guides by Kafle et al. [21, 39].

#### Experimental design and larval observation

**Fig. 2:**
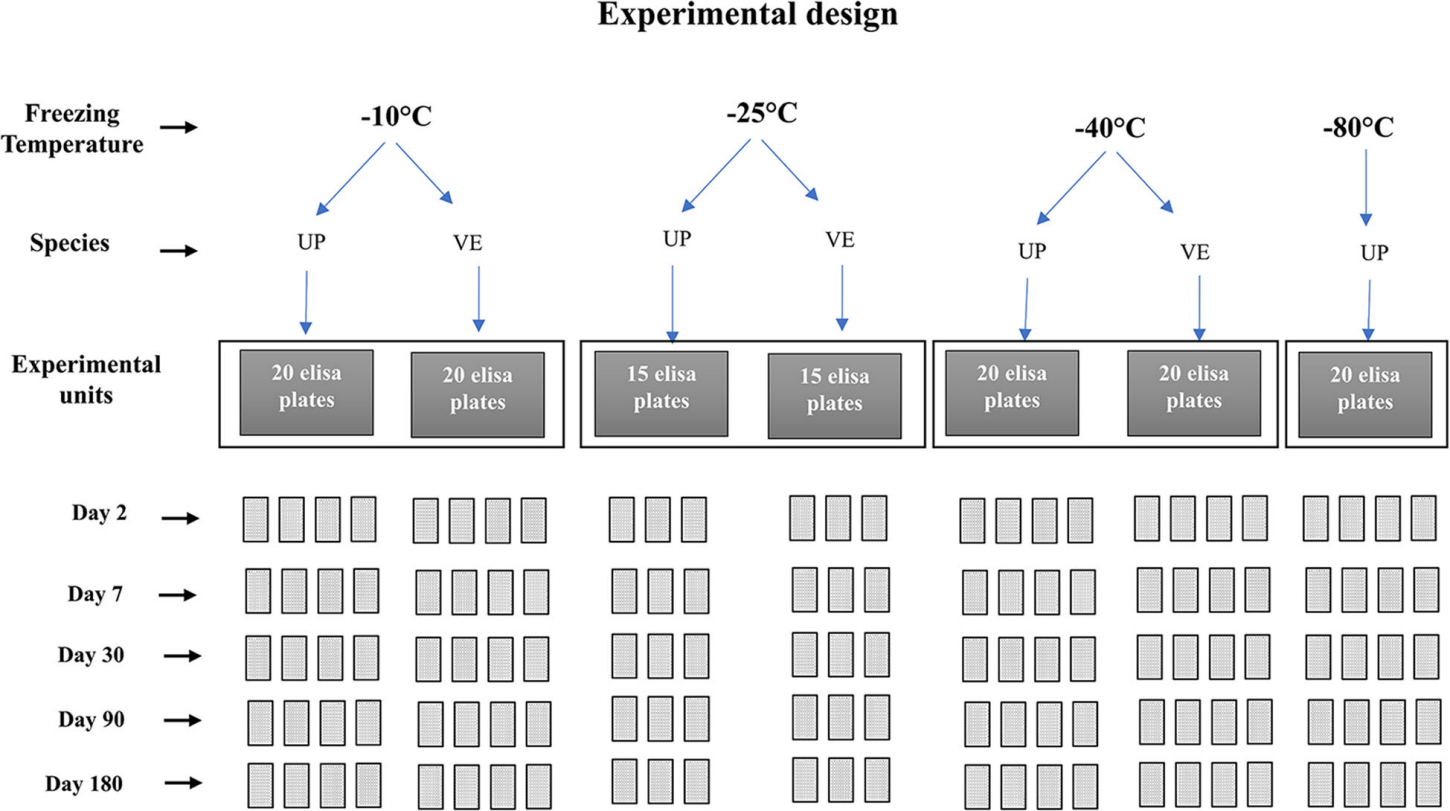
Experimental design for investigating the freezing survival of *Umingmakstrongylus pallikuukensis* (UP) and *Varestrongylus eleguneniensis* (VE). For each plate, L1 (1–4 L1/well) suspended in 200 μl tap water were placed in each of 40 wells, except at -25 °C where 2–10 L1/well were used

The freezing survival of *U. pallikuukensis* and *V. eleguneniensis* was studied under four (-10, -25, -40 and -80 °C) and three (-10, -25 and -40 °C) sub-zero temperatures, respectively (Fig. 2). Each temperature treatment comprised 15 or 20 ELISA plates (Eppendorf) for each species (Fig. 2), with each of 40 wells in an ELISA plate containing one to ten individual parasites suspended in 200 μl of tap water. Survival to 2, 7, 30, 90 and 180 days post-freezing was evaluated for both parasites (Fig. 2). Before subjecting to freezing, each well (labelled with a unique identification number) was observed under 400× magnification, the species identity was reconfirmed morphologically, and the number of the L1 present in each well was recorded. Only live L1 (motile larva) were considered for the experiment. Wells that had over 10 initial individuals were not included in the study because it was difficult to accurately assess the survival with such high density of L1. The ELISA plates for both species were placed in a Rubbermaid container in temperature-controlled freezers. The temperature inside each container was monitored every 15 minutes using LogTag recorder. On the day of observation, four plates of each species were selected randomly (three plates at -25 °C because of a shortage of L1), left to thaw at room temperature on the lab bench for one h, and then observed under 200× magnification. Any larvae that were deformed were considered not viable and thus recorded as dead. Larvae that did not show any motility within two minutes of observation were kept at 4 °C for another 24 h, and if they had not regained motility after 24 h, they were recorded as dead.

### Data analysis

All analyses were conducted using R statistical software [40]. The thermal parameters for larval development inside the intermediate host, i.e. the threshold temperature, temperature of theoretically zero development (T_0_), and degree-days for development (DD), were estimated by fitting the daily development rate $\frac{1}{D}$ to a simple linear equation for each temperature:

1 $$ \frac{1}{D}=\frac{T_0+T}{DD} $$

where *D* is the number of days from infection to the first appearance of intermediate third-stage larvae (iL3) and *T* is temperature. The parameters were estimated by linear regression of development rate (1/D) over temperature (*T*), where slope equals 1/DD and the intercept equals T_0_/DD [41, 42]. We fitted a linear model for our parameter estimates for three reasons: (i) linear model fitted our data well, (ii) our objective was to derive DD and T_0_, and (iii) we wanted to compare to other protostrongylids, especially with lungworm *U. pallikuukensis*, and linear models were used to derive the parameters for these protostrongylids.

Binomial generalized linear models (GLM; logit link) were fitted to investigate the effect of temperature and freezing duration on the survival of *U. pallikuukensis* and *V. eleguneniensis*. The response was the proportion of individuals in each well surviving until inspection at 2, 7, 30, 90 or 180 days freezing duration, calculated as the surviving L1s divided by the initial number of viable parasites in each well. Fixed effects included parasite species, temperature, freezing duration, and the interaction between temperature and species, representing the differential effect of freezing temperature on the two species. The plate was initially included as a random effect, but the variance among the plates was small, and the inclusion of the random effect did not change the parameter estimates for the fixed effects, so the effect of the plates was ignored. Ten different models were fitted representing different combinations of the four fixed effects. The models were compared using Akaike Information Criterion (AIC). There was a high degree of model and parameter uncertainty, and so we report the model-averaged predictions for survival over the top five models which comprised more than 90% of the cumulative Akaike weight [43], using the AICcmodavg library [44].

## Results

### Temperature-dependent development of *V. eleguneniensis*

**Fig. 3:**
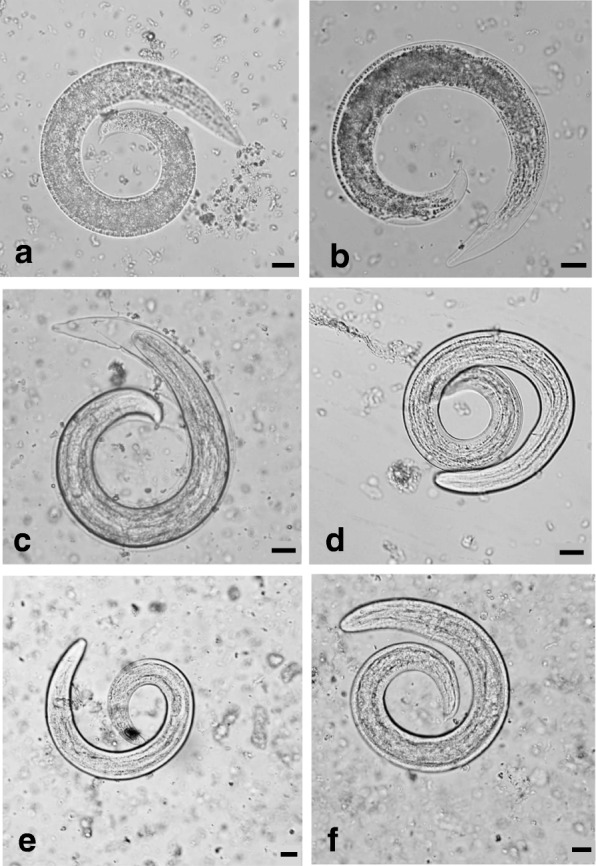
Developing larvae of *Varestrongylus eleguneniensis* at various days post-infection (dpi) at 12 °C when observed under 400× magnification. **a** First-stage larva at 40 dpi. **b** Second-stage larva at 47 dpi. **c** Early third-stage larva at 55 dpi. **d** Intermediate third-stage larva at 61 dpi. **e** Late third-stage larva at 67 dpi. **f** Emerged third-stage larva at 72 dpi. Larval stages were differentiated based on Kutz et al. [25] and Verocai et al. [20]. *Scale*-*bars*: 20 **μ**m

**Fig. 4:**
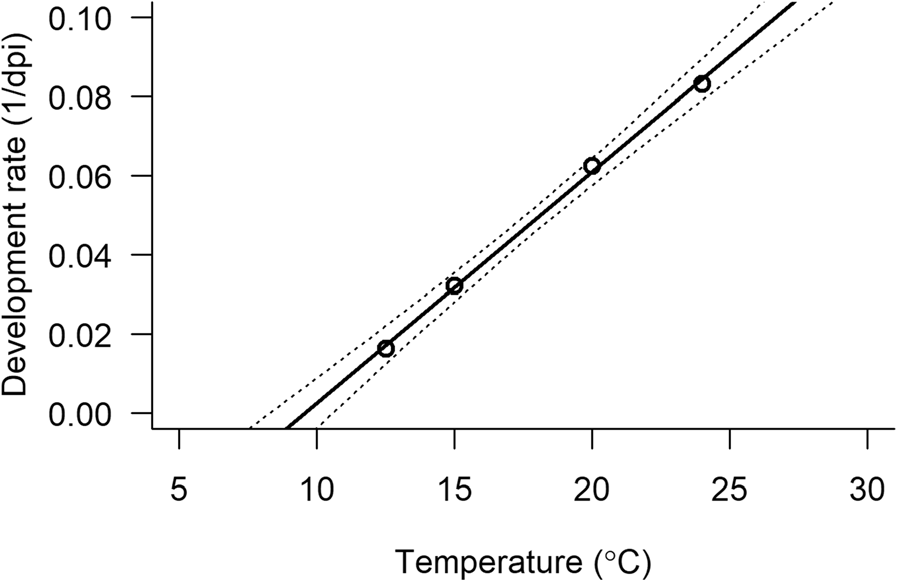
Development rate of *Varestrongylus eleguneniensis* in *Deroceras laeve* from 8.5 to 24 °C. White circles represent the daily development rates at the specific temperatures, the solid line represents the fitted linear regression curve, and the dashed line is the 95% confidence interval in model predictions

*Varestrongylus eleguneniensis* larvae successfully developed from L1 to L3 at temperatures between 12.5 to 24 °C (Table 1; Figs. 3 and 4. At 8.5 °C, L1 developed to L2 by 50 days post-infection (dpi), but no L3 were observed on weekly slug digestion by day 101. After day 101, the sampling interval was changed to lengthen the trial, and the remaining slugs were monitored, fed regularly, and the slugs that died were digested. The last slug was digested at 166 days and no L3 were detected. Development occurred faster at higher temperatures (Table 1, Fig. 4). Development rate increased significantly with temperature according to the equation (e.g., 1/dpi = -0.557 + 0.0058 T (*F*_(1, 2)_ = 1297, *P* < 0.0001), with *R*^2^ of 0.99. From equation 1, the threshold temperature was determined as T_0_ = 9.54 °C (95% CI: 8.25–10.57 °C; based on 95% CI on model predictions in Fig. 2) and DD was 171.25 (95% CI: 153–194), which are within the range determined for other northern protostrongylids (Table 2).

**Table 1:** Study design and results from the experimental infection of *Deroceras laeve* with *Varestrongylus eleguneniensis*

Exp. No.	Temperature (°C) (Mean ± SD)	Infection dose (L1/slug)	No. of slugs	Mean slug weight ± SD (g)	Slugs examined on digestion day	Days examined	Day first iL3^a^ observed	% L3-recovery^b^
1	8.5 ± 0.26	200	40	0.09 ± 0.04	1–3	50, 60, 67, 74, 81, 87, 94, 101, 124, 135, 166	ND^c^	–
2	12.5 ± 0.30	200	35	0.10 ± 0.04	3	40, 47, 52, 55, 57, 59, 61, 67	61	10.83
3	15.0 ± 0.30	200	35	0.10 ± 0.03	3	21, 22, 25, 26, 27, 29, 31, 32, 33, 34, 36	31	12.00
4	20.0 ± 0.29	300	35	0.12 ± 0.04	2–3	14, 15, 16, 17, 19	16	18.40
5	24.0 ± 0.32	200	35	0.10 ± 0.04	2–5	11, 12, 13, 14, 15, 35	12	13.80

^a^Days post-infection when at least one intermediate L3 was observed from any one of the slugs digested

^b^The percentage of larvae that potentially would have developed into infective L3. This was calculated by dividing the average number of larva per slug on the first day when the intermediate L3 was observed, with the infection dose (per slug) and multiplied by 100

^c^Not detected

**Table 2:** Development rates of nematodes within the protostrongylidae family

Protostrongylid	Intermediate host	Regression equation	*R* ^2^	T_0_ (°C)	DD	Reference
*Varestrongylus eleguneniensis*	*Deroceras laeve*	*y* = -0.0557 + 0.0058*x*	0.99	9.54	171	Present study
*Umingmakstrongylus pallikuukensis*	*Deroceras laeve*	*y* = -0.0510 + 0.0060*x*	0.97	8.50	167	[25]
	*Deroceras reticulatum*	*y* = -0.0570 + 0.0060*x*	0.99	9.50	167	[25]
*Parelaphostrongylus odocoilei*	*Deroceras laeve*	*y* = -0.0522 + 0.0060*x*	0.98	8.50	163	[26]
*Elaphostrongylus rangiferi*	*Arianta arbustorum*	*y* = -0.0410 + 0.0040*x*	0.99	10.25	250	[48]
	*Euconulus fulvus*	*y* = -0.0330 + 0.0040*x*	1	8.25	250	[48]
*Muellerius capillaris*	*Deroceras reticulatum* or *Deroceras agrestis*	*y* = -0.0250 + 0.0060*x*	0.96	4.20	167	[68]

In the pilot study on larval emergence, L3 emerged from four of the six slugs maintained individually at 12.5 °C from day 62 to day 87. Two slugs died at day 68, and no L3 had emerged from these slugs up to that point. For the remaining four slugs, larval emergence was first observed at 70 dpi (two slugs) and 74 dpi (two slugs). Emergence from all four slugs continued to day 83, and although no emergence was detected on subsequent observations, L3 were found inside all of the slugs on digestion after they died at days 85 and 87, respectively.

### Freezing survival of *U. pallikuukensis* and *V. eleguneniensis* L1

**Fig. 5:**
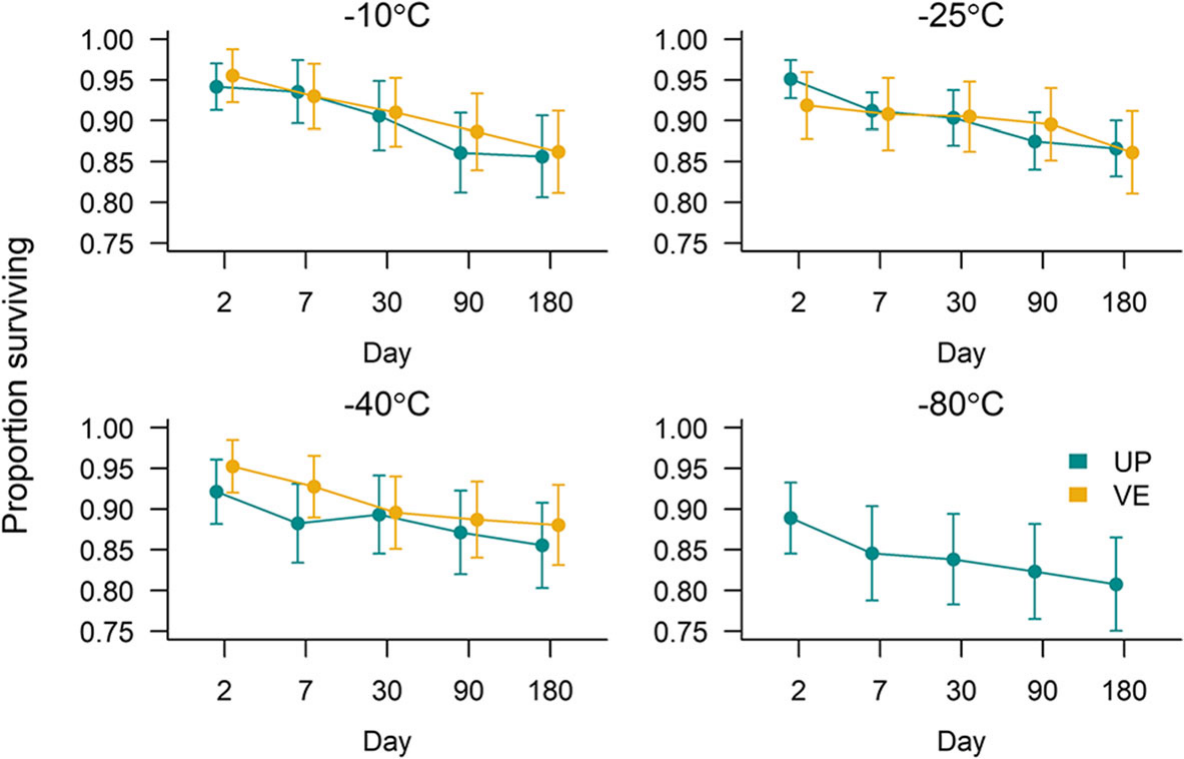
Freezing survival of *U. pallikuukensis* (UP) and *V. eleguneniensis* (VE) at four sub-zero temperatures and different freezing durations. The proportion surviving was calculated as the total number of L1 surviving over the total number of initially viable L1 in all wells for that species, temperature, and day combination. The error bars represent the 95% confidence interval on the proportion, calculated as p ± 1.96*[p*(1-p)/i] where *p* is the proportion, and *i* is the initial viable L1. An experiment could not be performed for *V. eleguneniensis* at -80 °C because insufficient larvae of this species were available

**Fig. 6:**
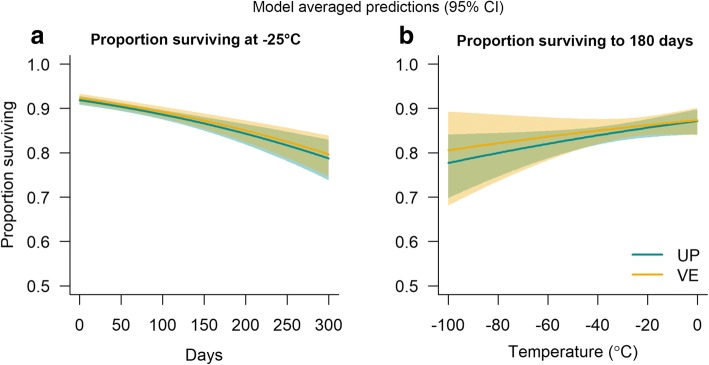
Model-averaged predictions (Table 2) for the survival of *Umingmakstrongylus pallikuukensis* (UP) and *Varestrongylus eleguneniensis* (VE) relative to freezing duration at -25 °C (**a**) and relative to temperature if frozen for six months (**b**)

L1 of both *U. pallikuukensis* and *V. eleguneniensis* had high freezing survival at all temperatures and all durations (Fig. 5). There was a high degree of model uncertainty, with five of the ten models tested making up 90% of the cumulative Akaike weight (Table 3). Temperature and freezing duration were fixed effects in all five top models and, therefore, were more important drivers of survival than species (Table 3). Survival decreased with increasing freezing duration and decreasing temperature in the top five models (Table 4). Despite three of the top models including an effect of species (Table 2), within each model, the parameter estimate(s) for species were not significantly different from zero (Table 4), suggesting the effect of species was weak and freezing survival of *U. pallikuukensis* and *V. eleguneniensis* L1 was similar (Fig. 6). The model-averaged predicted time for 50% mortality of L1 when kept at -25 °C was 653 days (95% CI: 618–677) for *U. pallikuukensis* and 668 days (95% CI: 631–695) for *V. eleguneniensis*.

**Table 3:** Summary of the models (binomial GLM, logit link) fitted to experimental data on the survival of *U. pallikuukensis* and *V. eleguneniensis*, including effects of freezing duration (day), temperature (temp), and parasite species (species). Only the top 5 models comprising 90% of the cumulative Akaike weight were considered

Predictors	Model number	K^a^	AIC	Δ_*i*_^b^	*w*_*i*_^c^	cum *w*_*i*_^d^
Day + temp	1	3	4182.196	0	0.204	0.204
Day*temp^e^	2	4	4182.276	0.080	0.196	0.401
Day + temp*species^e^	3	5	4182.407	0.211	0.183	0.584
Day + temp + species	4	4	4182.617	0.421	0.165	0.750
Day*temp^e^ + species	5	5	4182.648	0.452	0.163	0.913
Day*species^e^ + temp	6	5	4184.53	2.334	0.064	0.976
Day*temp*species^e^	7	8	4186.516	4.320	0.023	0.999
Day + species	8	3	4198.767	16.571	5.15E-05	0.999
Day*species^e^	9	4	4200.717	18.521	1.94E-05	0.999
Day	10	2	4202.291	20.095	8.84E-06	1

^a^K = number of parameters.

^b^Δ_*i*_ = AIC – min(AIC)

^**c**^Akaike weights: $$ {w}_i=\exp \left(-0.5{\Delta}_i\right)/\sum \limits_j\exp \left(-0.5{\Delta}_j\right) $$

^d^Cumulative Akaike weight

^e^and *, interactive and additive effects included

**Table 4:** Parameter estimates on the scale of the linear predictor from the top models (Table 3)

Model	Explanatory variables	Estimate	SE	Adjusted *w*_*i*_^a^	Cum. adj. *w*_*i*_
1	Intercept	-2.63932	0.08059	0.22	0.22
	Day	-0.00359	0.00053		
	Temp	0.00822	0.00171		
2	Intercept	2.72400	0.01016	0.22	0.43
	Day	-0.00472	0.00097		
	Temp	0.01066	0.00244		
	Day:temp	0.00003	0.00002		
3	Intercept	2.61770	0.09395	0.20	0.63
	Day	-0.00359	0.00053		
	Temp	0.00852	0.00185		
	SpeciesVE	-0.12535	0.17512		
	Temp:speciesVE	-0.00860	0.00578		
4	Intercept	2.58468	0.09098	0.18	0.82
	Day	-0.00360	0.00053		
	Temp	0.00764	0.00176		
	SpeciesVE	0.10576	0.08447		
5	Intercept	2.66900	0.10970	0.18	1
	Day	0.00474	0.00097		
	Temp	0.01009	0.00247		
	SpeciesVE	0.10750	0.08453		
	Day:temp	0.00003	0.00024		

^a^Adjusted *w*_*i*_ is calculated using the formula from Table 3, but only including the five top models in the denominator *j*

## Discussion

Typical to any protostrongylid, the rate of larval development for *V. eleguneniensis* inside the intermediate host, *D. laeve*, was positively related to temperature, and the lower threshold temperature (T_0_) and the thermal constant required for development into infective stage (DD) were within the range determined for other northern protostrongylids (Table 2). We found that *D*. *laeve* is a good intermediate host for *V. eleguneniensis*, and the observed larval establishment and L3 recovery were substantially better than observed in our laboratory for the common garden slug, *D. reticulatum* [38]*.* On Victoria Island, where the range of *V. eleguneniensis* is rapidly expanding, *D. laeve* is the only suitable slug intermediate host that has been documented [35], and a high rate of larval uptake may be important for successful transmission. The evidence of larval emergence, albeit in a pilot study, supports the possibility of an ecological adaptation by *V. eleguneniensis* to allow overwinter transmission, as described for *U. pallikuukensis* and, to a lesser extent, for *P. odocoilei* [26, 27]. The high freezing survival of both *U. pallikuukensis* and *V. eleguneniensis* L1 not only provides new information on the cold hardiness of these parasites, but quantifies these parameters for use in parasite transmission models. The rate of larval survival as a function of freezing temperature and duration is also important for designing cryopreservation strategies and estimating the larval survival in frozen samples. The similar ability of *U. pallikuukensis* and *V. eleguneniensis* to survive freezing for the extended period is intriguing, as *U. pallikuukensis* being a relatively specialized Arctic parasite, was expected to survive better than *V. eleguneniensis*, a parasite more broadly distributed across the sub-Arctic.

Comparing the thermal requirements and freeze tolerance between *V. eleguneniensis* and *U. pallikuukensis* is important in the context of their differential rates of range expansion in the Canadian Arctic Archipelago. Field surveys suggest slower colonization of *V. eleguneniensis* compared to *U. pallikuukensis*, despite the former being a multi-host parasite of caribou, muskoxen, and moose with a greater dispersal potential with migrating caribou [21], compared to the latter, which is specific to muskoxen, a non-migratory species. While various ecological and epidemiological factors might influence the transmission dynamics of these parasites, their thermal requirements and overwinter survival, and those of the gastropod intermediate hosts, probably dictate their northern range limit. All else being equal, parasite species with lower T_0_ and DD are likely to expand their range more quickly and establish at a more northern latitude. Similarly, the parasite with greater L1 freeze tolerance has survival advantages at higher latitudes. Our findings of higher T_0_ and DD of *V. eleguneniensis* compared to *U. pallikuukensis* [25] are consistent with their differential range expansion. As *U. pallikuukensis* and *V. eleguneniensis* do not differ in their ability to survive freezing, it is unlikely that freeze survival is contributing to the differential range expansion. In light of our findings and the preliminary results on the distribution of these parasites, it can be hypothesized that higher thermal requirements are contributing to the slower northward spread of *V. eleguneniensis* compared to *U. pallikuukensis*. This can be tested and validated by incorporating the parameters into models that determine the fundamental thermal niche for these parasites. However, our previous work, experimental studies and broad-based surveys suggest that *U. pallikuukensis* is much more fecund than *V. eleguneniensis* ([38, 45], Kafle, Kutz unpublished data). This may also contribute to more rapid range expansion of the former parasite. Other life-history traits such as infectivity, host abundance, and their interactions, are also important in the colonization success of invading parasites [17, 46, 47] and likely play a significant role here as well.

The developmental parameters and freeze tolerance capabilities among protostrongylids with Arctic and sub-Arctic distributions are quite comparable - developmental thresholds lie around T_0_ = 8 to 10 °C and L1 are very resistant to lethal effects of freezing [29–32]. Within the narrow range, however, T_0_ vary among parasite species and among intermediate hosts within a parasite species (Table 2). For instance, *Elaphostrongylus rangiferi*, a common parasite of wild and semi-domestic reindeer in the Palaearctic region and woodland caribou of Newfoundland, Canada, and *U. pallikuukensis*, a sympatric protostrongylid, have different temperature thresholds in different species of intermediate hosts [25, 48]. Based on two studies, the thermal requirement for development to L3 (DD), however, seems to be constant within a parasite species regardless of the intermediate host; DD is the same in different intermediate host species for *U. pallikuukensis* and *E. rangiferi* [25]. The literature on the freezing survival of northern protostrongylids is scarce, but based on at least two well-designed studies it is clear that northern protostrongylids are highly cold tolerant. Shostak & Samuel [29] reported over 90% survival of *P. odocoilei* L1 suspended in water and frozen at -25 °C for 280 days. In another study, Lorentzen & Halvorsen [49] did not observe reduced survival of *E. rangiferi* L1 when frozen with water at -20 °C for 360 days, and a similar survival pattern was observed for L1 (in the feces) at -80 °C, frozen for a similar time. Developmental thresholds and freezing survival of more temperate species seem to be comparatively lower. For example: *Muellerius capillaris*, a temperate protostrongylid, has a considerably lower T_0_ (4.2 °C) and is less tolerant to freezing despite being relatively closely phylogenetically related to *U. pallikuukensis* [50]. *Parelaphostrongylus tenuis*, another temperate species, also has a lower freezing tolerance than it northern counterparts - approximately 70% survival of L1 when frozen in feces at -15 to -20 °C for 182 days [32]. In another study, Forrester & Lankester [30] found that 76% of *P. tenuis* L1 survive a constant temperature of -14 °C in the lab when frozen in fecal pellets for four months. The variation in developmental and survival parameters among protostrongylid species is most likely a result of biological differences between both parasite and host species that are shaped by various ecological and evolutionary processes [25, 50]. For instance, it has been suggested that higher T_0_ for northern protostrongylids is an ecological adaptation to the Arctic conditions (e.g. long winter, short transmission period) [51]. Higher T_0_ ensures that L1 enter the developmental phase only when the temperature is consistently warm, thereby increasing the chances of successful development to L3 in a single season. This further prevents mortality of developing larvae (survival of L1 is higher than L2 and L3 in overwintering intermediate host) and the intermediate hosts themselves over the winter [51].

The extreme freeze tolerance of *U. pallikuukensis* and *V. eleguneniensis* warrants further explorations on mechanisms of freeze resistance. For other nematodes, several biochemical and physiological mechanisms to survive freezing have been identified [8, 52–54]. Biochemical mechanisms include the synthesis of different proteins (commonly called antifreeze proteins) or cryoprotectants (e.g. trehalose, glycerol; [9, 55, 56]. Physiological mechanisms include the behavioral strategies to resist the lethal effects of freezing, which can be broadly classified into three types: (i) freeze avoidance (enables body fluid to remain liquid at temperature below their melting point); (ii) freeze tolerance (surviving some degree of ice nucleation in the body); or (iii) cryoprotective dehydration (desiccation at low temperatures to prevent freezing) [9]. We do not know the freezing survival strategy used by *U. pallikuukensis* and *V. eleguneniensis* L1, but because the survival was very high in water, they may employ a freeze-tolerance strategy and survive inoculative freezing, as described for other nematodes [52, 57, 58].

## Conclusions

The Arctic continues to warm at an unprecedented rate, driving the emergence and spread of pathogens [19, 59, 60] and thereby escalating the threats on the sustainability of native Arctic wildlife [61–63]. Since healthy wildlife is critical for food safety and security in the northern communities, it is vital to understand and anticipate future trends in emerging diseases in order to devise proactive management plans. Ecological models parameterized with physiological data capture the mechanisms behind observed patterns of distribution and abundance, and are particularly useful as predictive frameworks for investigating an organism’s response to climate change [16, 64–67]. Our study provides key development and survival parameters of two parasites that are undergoing rapid range expansion in the Canadian Arctic, and these parameters can be incorporated into mechanistic models to describe and forecast the climate-driven range expansion of these parasites, as well as to understand the current and future trends of the infection dynamics. Predicting changes in disease dynamics and wildlife health under unprecedented climate change requires experimental studies such as ours to elucidate organisms’ ecophysiology in order to parameterize mechanistic ecological models [18, 33].

## Abbreviations

T_0_: Lower development threshold
DD: Development degree-days
dpi: Days post-infection
CI: Confidence interval
AIC: Akaike information criterion
iL3: Intermediate third-stage larvae
SD: Standard deviation
SE: Standard error
