# Correction to: Temperature-dependent development and freezing survival of protostrongylid nematodes of Arctic ungulates: implications for transmission

**DOI:** 10.1186/s13071-018-3029-8

**Published:** 2018-08-02

**Authors:** Pratap Kafle, Stephanie J. Peacock, Sarah Grond, Karin Orsel, Susan Kutz

**Affiliations:** 1 Faculty of Veterinary Medicine, University of Calgary, Calgary, AB Canada; 2 Department of Biological Sciences, Faculty of Science, University of Calgary, Calgary, AB Canada; 3 Department of Ecology and Evolutionary Biology, Princeton University, Princeton, NJ USA

## Correction

Unfortunately, the original version of this article [1] contained an error. In Fig. 1, the longitude scale of the map should not have the negative sign. Figure 1 has been corrected in the original article and is also included correctly below. The publisher apologizes for the inconvenience caused.

**Fig. 1:**
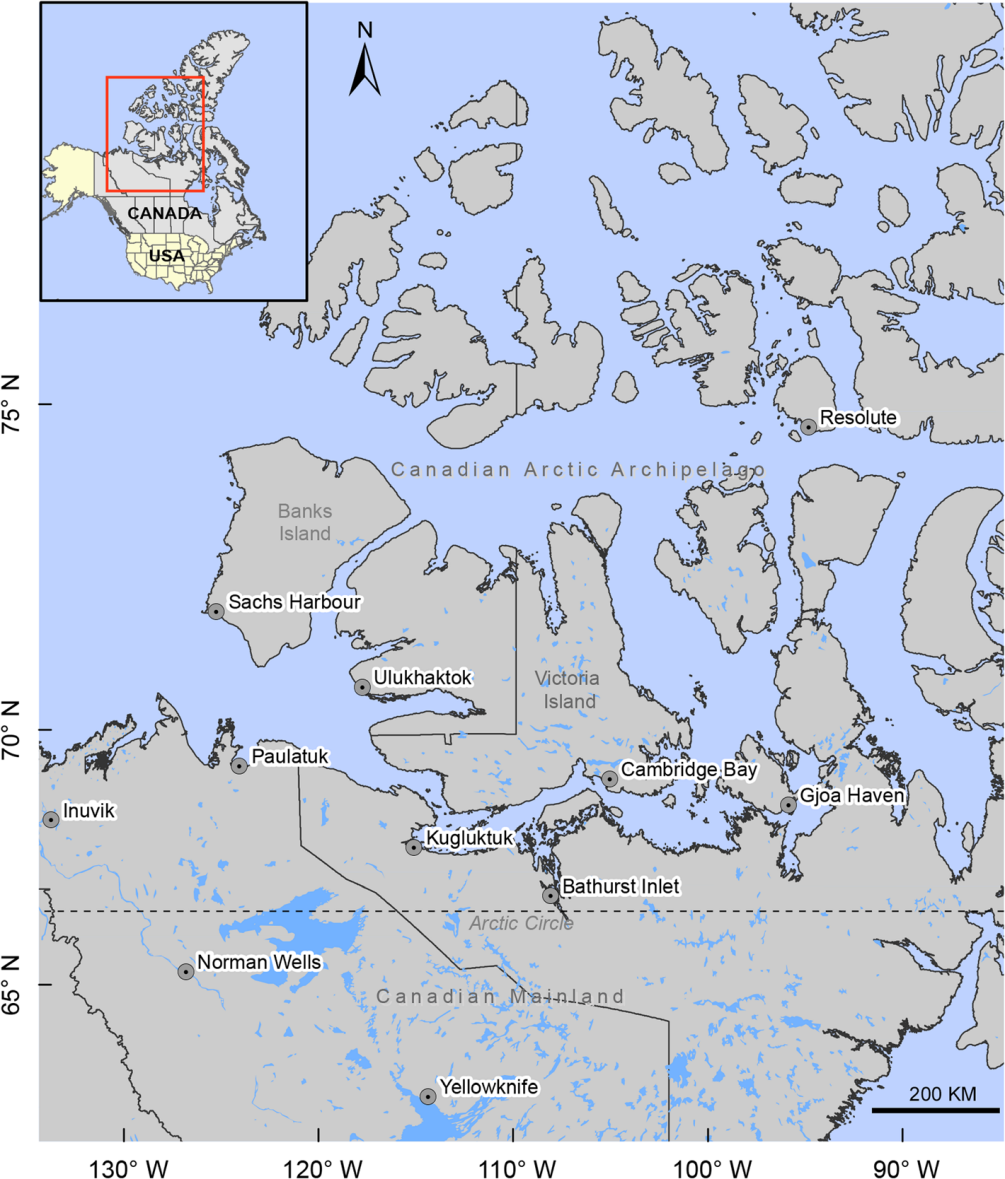
Map showing the arctic mainland Canada and Canadian Arctic Archipelago
